# XGBoost–SHAP Interpretable Modeling Identifies and Validates an Eight-Gene Biomarker for Hepatic Encephalopathy Risk Prediction in Cirrhosis

**DOI:** 10.3390/ijms27156925

**Published:** 2026-08-01

**Authors:** Yuanfeng Lan, Tian Zhao, Ying Xu, Haihong Ye

**Affiliations:** 1 Department of Medical Genetics and Developmental Biology, School of Basic Medical Sciences, Capital Medical University, Beijing 100069, China; yuanfeng_lan@mail.ccmu.edu.cn (Y.L.); zhaotian@ccmu.edu.cn (T.Z.); 2 Laboratory for Clinical Medicine, Capital Medical University, Beijing 100069, China; 3 Beijing Key Laboratory of Cell and Gene Therapy in Otology, Beijing 100069, China; 4 Department of Human Cell Biology and Genetics, SUSTech Homeostatic Medicine Institute, School of Medicine, Southern University of Science and Technology, Shenzhen 518055, China

**Keywords:** cirrhosis, hepatic encephalopathy, machine learning, prediction model, prognostic analysis

## Abstract

Cirrhosis, accounting for 2.4% of global mortality in 2019, represents a leading cause of death in chronic liver disease. Hepatic encephalopathy (HE), a decompensated complication of cirrhosis, is associated with a median survival of only 0.92 years post-diagnosis. Current screening methods relying on neuropsychological tests (e.g., Psychometric Hepatic Encephalopathy Score, PHES) have limitations such as time-consuming procedures and subjective interpretation, potentially delaying diagnosis. To address this, we integrated four cirrhotic transcriptomic cohorts (GSE41919, GSE57193, GSE139602, and GSE15654) and employed an integrated algorithm (LASSO [Least Absolute Shrinkage and Selection Operator]–RFE [Recursive Feature Elimination]–random forest) to identify HE-specific biomarker genes. Ultimately, we developed an HE risk-prediction system centered on eight HE-specific marker genes, namely, *PRB2*, *TUBA1C*, *NPC2*, *LRRC32*, *TLN1*, *SOX9*, *SERPINA3* and *RNASE4*. Based on these genes, an XGBoost (eXtreme Gradient Boosting)-based HE risk stratification model was constructed, and SHAP (SHapley Additive exPlanations) analysis was further introduced to address the “black-box” limitation of conventional machine learning models and to improve the interpretability. The finalized eight-gene system enables accurate, efficient, and interpretable HE risk assessment in patients with cirrhosis. Functional characterization through gene set enrichment analysis and structural equation modeling further revealed that these marker genes converge on four interconnected biological processes, namely, metabolic homeostasis, synaptic and neural transmission, immune inflammatory signaling, and hepatic detoxification, which collectively reflect the gut–liver–brain axis disruption central to HE pathogenesis. This dual-model system, incorporating both cirrhosis progression and survival prognosis, provides a reliable and clinically applicable tool for early HE risk warning and stratification, reducing the limitations of traditional neuropsychological screening and offering a translational foundation for timely intervention and prognostic optimization in high-risk cirrhotic patients.

## 1. Introduction

Cirrhosis, a leading cause of chronic liver disease-related mortality, has seen rising incidence and death rates since 1990, accounting for 2.4% of global deaths in 2019 [1,2,3]. Clinically, it progresses from a compensated to a decompensated phase, the latter presenting as hepatic encephalopathy (HE), ascites, or variceal hemorrhage [4,5]. Among these, HE stands out as it reflects cirrhosis progression and independently predicts poor survival, with a median post-diagnosis survival of only 0.92 years [6,7]. Approximately 40% of cirrhosis patients remain undiagnosed until a decompensating event occurs [6]; early detection could significantly improve both survival and quality of life [8,9].

HE presents a spectrum of symptoms ranging from mild cognitive impairment (minimal hepatic encephalopathy, MHE) to coma. Because its manifestations are nonspecific, the condition is often misdiagnosed or overlooked [10]. Current practice relies on neuropsychological tests, such as the Stroop test and Psychometric Hepatic Encephalopathy Score (PHES), for MHE screening, especially in high-risk patients with comorbid depression [11]. These tests are time-consuming and require specialized training, limiting their use in routine care [12]. Overlap between HE symptoms and other cirrhosis comorbidities (depression, uremic encephalopathy) compounds the diagnostic difficulty [10,13,14]. Early detection could slow disease progression and improve outcomes of cirrhosis, and yet current screening methods fall short of this goal. Therefore, molecular biomarker-based early warning systems are needed for clinical deployment.

In recent years, machine learning methods have been increasingly applied in chronic liver disease and hepatocellular carcinoma outcome prediction, representing a rapidly growing area of research. Numerous studies have focused on developing predictive models using routine clinical data to identify high-risk patients and guide early intervention. For instance, Grubert Van Iderstine et al. conducted a large-scale retrospective cohort study developing and validating an optimized XGBoost model to predict 1-, 3-, 5-, and 10-year hepatic decompensation risk at the time of the first outpatient visit in cirrhotic patients [15]. An et al. developed a multicenter model that integrated natural language processing for automated electronic health record data extraction with an XGBoost algorithm to predict prognosis in intermediate-stage HCC patients undergoing transarterial chemoembolization (TACE) [16]. Zhu et al. developed and validated an XGBoost-based machine learning model using large-scale population cohort data, demonstrating that the model could screen individuals with significant liver fibrosis risk in the general population, using easily accessible demographic and clinical indicators, with the risk score significantly correlated with all-cause mortality [17]. Collectively, these studies underscore the potential of machine learning, particularly XGBoost, for enabling early identification of high-risk individuals across the full spectrum of chronic liver disease, from fibrosis screening to decompensation prediction and post-treatment prognosis. However, despite these advances, an interpretable machine learning model specifically tailored for HE risk prediction in cirrhotic patients remains lacking.

To address this gap, we integrated transcriptomic data from multiple cirrhotic cohorts and built an interpretable XGBoost–SHAP machine learning model with cross-validation. The model predicts HE risk using an eight-gene marker (*PRB2*, *TUBA1C*, *NPC2*, *LRRC32*, *TLN1*, *SOX9*, *SERPINA3*, and *RNASE4*), and SHAP values trace each prediction to individual gene contributions—making the output interpretable enough for clinical use. By relying on transcriptomic markers rather than clinical manifestations, the system can flag risk before overt symptoms appear.

## 2. Results

### 2.1. Workflow of the Study

**Figure 1:**
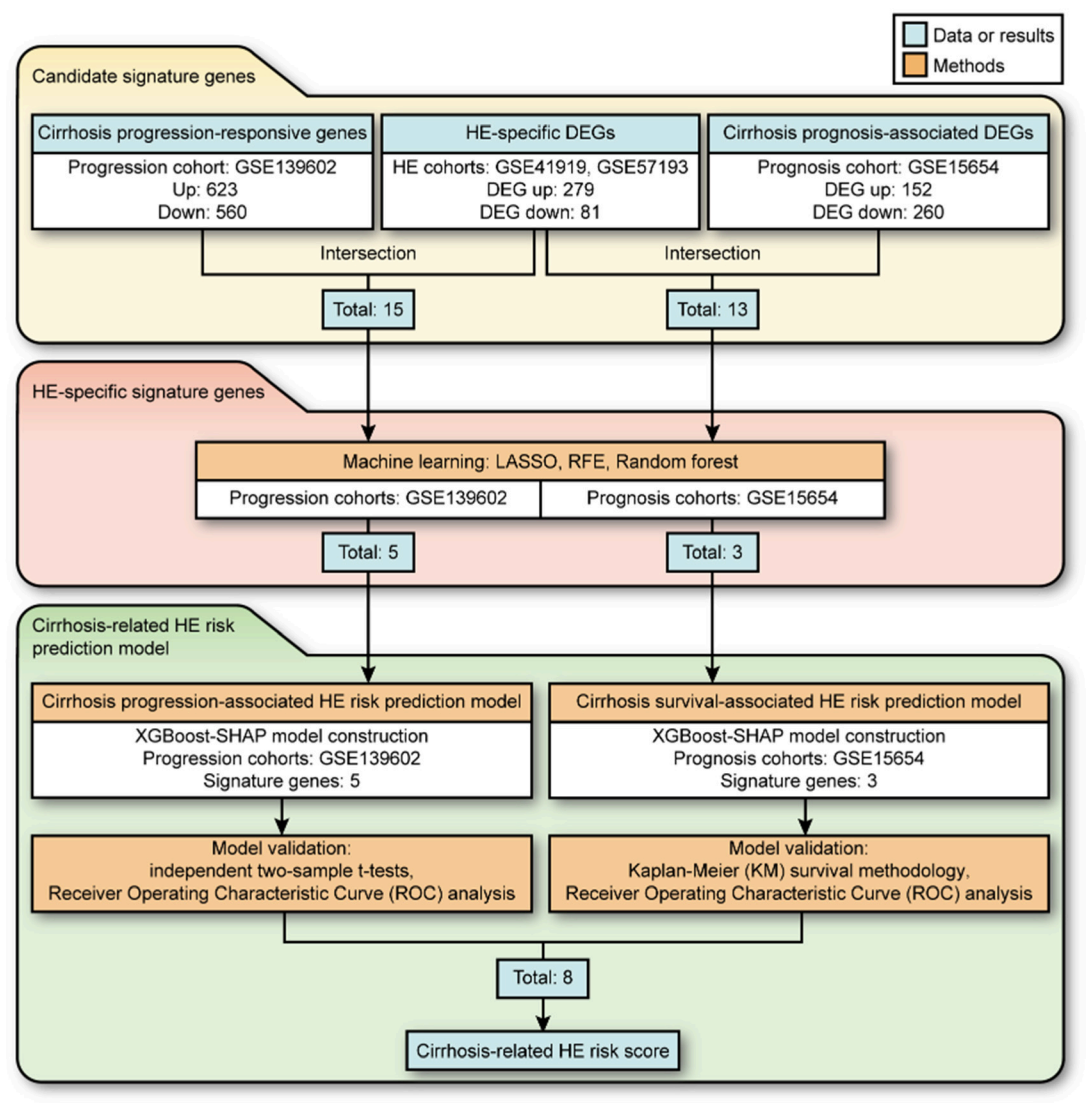
The flowchart of this study.

In this study, we leveraged four curated GEO datasets (GSE41919, GSE57193, GSE139602, and GSE15654) to identify HE-specific marker genes to establish an HE risk-prediction framework for cirrhosis (Figure 1 and Table 1). Our study employed the following trio as its analytical approach: (1) identification of HE-specific differentially expressed genes (DEGs) in two independent HE cohorts (GSE41919: three cirrhosis samples without HE vs. eight cirrhosis samples with HE; GSE57193: four cirrhosis samples without HE vs. four cirrhosis samples with HE); (2) identification of cirrhosis progression-reflecting genes in the progression cohort (GSE139602: six healthy controls, five early chronic liver disease cases [eCLD], 12 compensated cirrhosis specimens [CC], eight decompensated cirrhosis samples [DC], and eight acute-on-chronic liver failure samples [ACLF]); and (3) identification of cirrhosis prognosis-associated DEGs using a well-annotated prognosis cohort (GSE15654: *n* = 216 cirrhotic patients) (Figure S1). Overlapping genes in the above three analyses were then subjected to machine learning to select HE-specific marker genes for XGBoost–SHAP model construction and validation (Figure 1).

### 2.2. Identification of HE-Specific DEGs

**Figure 2:**
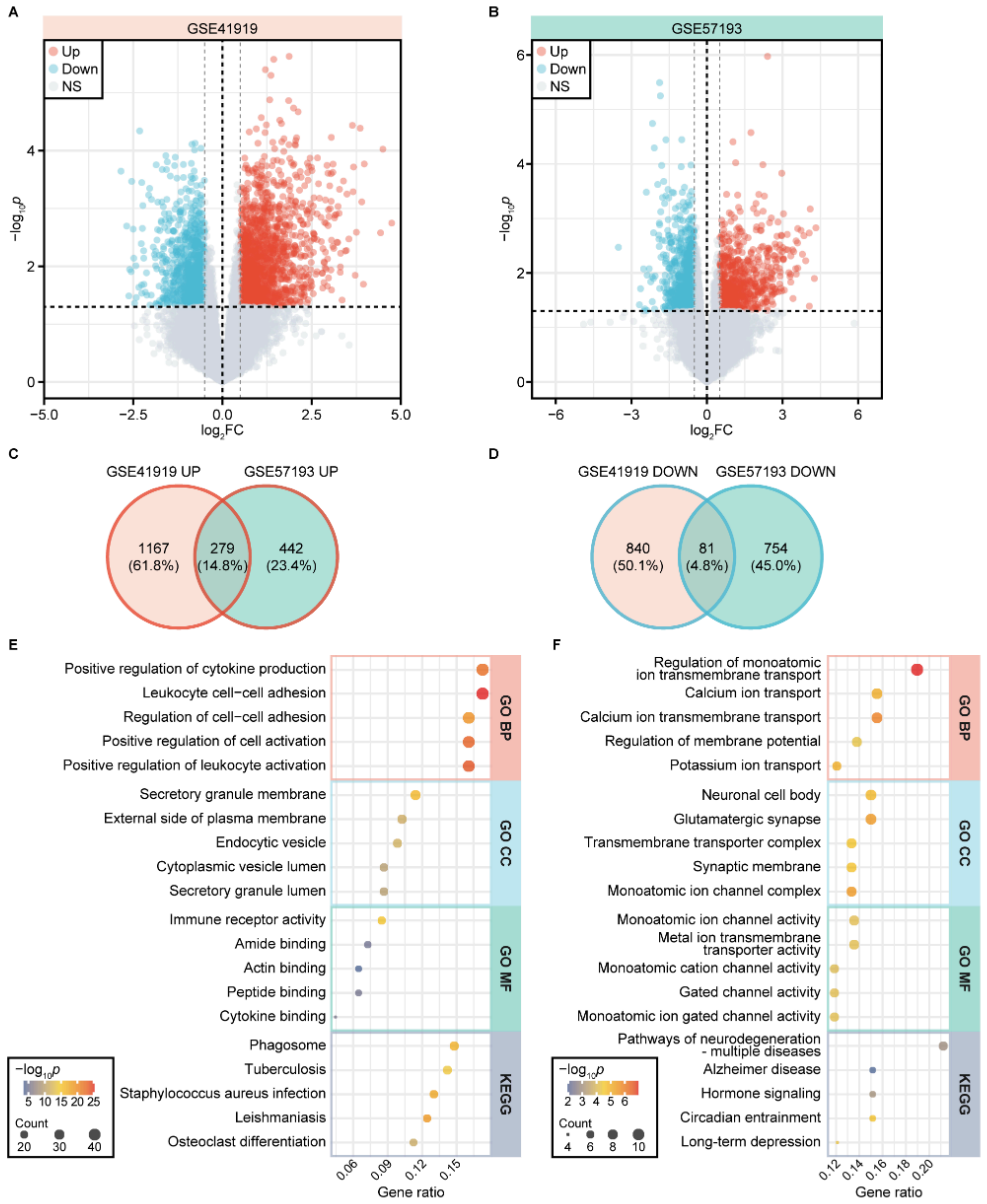
Identification and functional enrichment of differentially expressed genes (DEGs) in cirrhosis-associated HE. (**A**,**B**) Volcano plots of DEGs in GSE41919 (**A**) and GSE57193 (**B**) cohorts (threshold: |log_2_FC| ≥ 0.5 and *p* < 0.05), with red/blue dots representing up-/down-regulated genes, respectively. (**C**,**D**) Venn diagrams showing intersection analysis identifying 279 co-upregulated (**C**) and 81 co-downregulated (**D**) genes between cohorts. (**E**,**F**) Top 5 enriched GO BP, GO CC, GO MF and KEGG pathways for co-DEGs, with up-regulated genes (**E**) and down-regulated genes (**F**).

First, differential expression analysis was performed on the two HE cohorts (samples with vs. without HE) using the limma package (v3.64.3) on normalized expression metrices (*p*-value < 0.05 and |log_2_FC| ≥ 0.5), yielding 1446 upregulated and 921 downregulated DEGs in cohort GSE41919, and 721 upregulated and 835 downregulated DEGs in cohort GSE57193 (Figure 2A,B; Table S1). Intersection analysis identified 279 consistently upregulated (14.8% of the total upregulated genes) and 81 consistently downregulated DEGs (4.8% of the total downregulated genes) in both cohorts (Figure 2C,D; Table S1), establishing a reliable foundation for further investigation.

Having established a core set of HE-associated transcriptional changes, we next sought to understand their biological significance. To this end, we performed GO (Gene Ontology) and KEGG (Kyoto Encyclopedia of Genes and Genomes) enrichment analyses on the 279 upregulated and 81 downregulated DEGs. For upregulated DEGs, significant enrichment occurred in cell immunity-related pathways (Figure 2E, Table S2), including positive regulation of cytokine production (GO:0001819), leukocyte cell–cell adhesion (GO:0007159), positive regulation of leukocyte activation (GO:0002696), and phagosomes (hsa04145). This coordinated enrichment profile reflects systemic immune activation in HE during cirrhosis and is closely aligned with chronically inflammatory cascades in cirrhotic microenvironments [18].

For downregulated DEGs, significant enrichment was observed in pathways governing transmembrane transport dysregulation, membrane potential homeostasis, ion channel functionality, synaptic integrity, and neurodegenerative pathogenesis (Figure 2F), specifically encompassing regulation of ion transmembrane transport (GO:0034765), calcium ion transport (GO:0006816), calcium ion transmembrane transport (GO:0070588), regulation of membrane potential (GO:0042391), potassium ion transport (GO:0006813), Alzheimer disease (hsa05010), and pathways of neurodegeneration–multiple diseases (hsa05022). Collectively, changes in these pathways suggest significant impairment in neural transmission dynamics among HE patients.

### 2.3. Identification of Cirrhosis Progression-Reflecting Genes and Associations with HE

**Figure 3:**
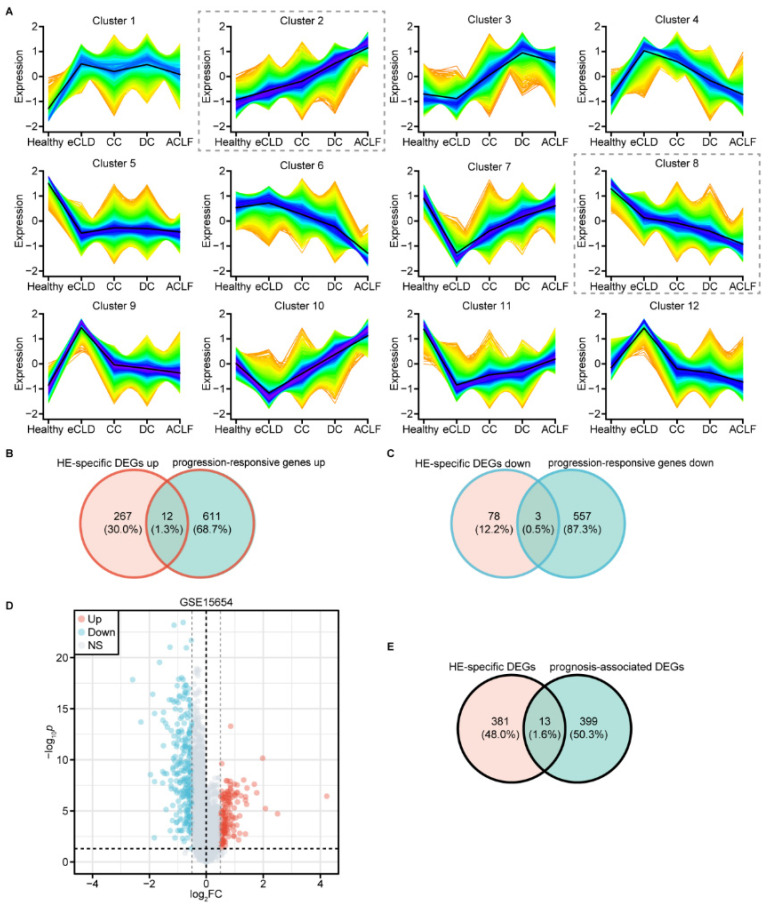
Screening and characterization of progression-responsive genes and prognosis-related DEGs. (**A**) Mfuzz clustering analysis revealing 12 gene expression modules, with dashed boxes highlighting modules showing sustained upward or downward trends. The color gradient from red to blue indicates the membership score of each gene to its assigned cluster, with red representing lower membership and blue representing higher membership. (**B**,**C**): Intersection analysis between HE-specific genes and progression modules, identifying 12 upregulated (**B**) and 3 downregulated (**C**) key genes. (**D**) Volcano plot of prognosis-related DEGs in GSE15654 cohort (412 genes, |log_2_FC| ≥ 0.5 and *p* < 0.05), with red/blue indicating up-/down-regulation. (**E**) Intersection analysis identifying 13 overlapping genes from DEGs related to HE and cirrhosis prognosis.

While the above analysis identified genes linked to the presence of HE, we next aimed to pinpoint genes whose expression dynamically reflects the progression of cirrhosis itself. To this end, we performed trajectory analyses using the Mfuzz algorithm (v2.62.0) [19] on the progression cohort (GSE139602) (Figure 1), which enables the identification of gene clusters with distinct expression patterns across disease stages. With the application of a module-progression correlation threshold, as r > 0.4, 12, distinct gene expression clusters were identified. Among these, cluster 2 (monotonically increasing, containing 623 upregulated genes) and cluster 8 (monotonically decreasing, 560 downregulated genes) emerged as pivotal progression-reflecting modules (Figure 3A and Table S1). The list of the 12 co-expression clusters is provided in the third sheet of Table S1.

To further explore the association of genes in clusters 2 and 8 with cirrhosis progression, we conducted GO and KEGG functional enrichment analyses, respectively. The 623 upregulated genes in cluster 2 were significantly enriched in extracellular matrix, cytoskeleton, and virus infection-related pathways, specifically, actin filament organization (GO:0007015), extracellular matrix organization (GO:0030198), actin binding (GO:0003779), cadherin binding (GO:0045296), PI3K-Akt signaling pathway (hsa04151), cytoskeleton in muscle cells (hsa04820), and human papillomavirus infection (hsa05165; Figure S2A and Table S3). Notably, significant enrichment of the upregulated genes within the PI3K-Akt signaling pathway (hsa04151) is consistent with the important role of this pathway in cirrhosis-driven oncogenic transformation [20,21].

In contrast, the 560 downregulated genes are significantly enriched in biological pathways orchestrating fundamental metabolic processes, neurodegenerative pathogenesis, and metabolic dysregulation syndromes (Figure S2B and Table S3), exemplified by purine-containing compound biosynthetic process (GO:0072522), small-molecule catabolic process (GO:0044282), purine nucleotide metabolic process (GO:0006163), pathways of neurodegeneration-multiple diseases (hsa05022), Alzheimer disease (hsa05010), and non-alcoholic fatty liver disease (hsa04932). Taken together, these results suggest that cirrhosis progression critically impairs hepatic metabolic homeostasis and likely exacerbates neurodegenerative cascades via systemic metabolic perturbations.

Through intersection analysis of the HE-specific DEGs (Figure 2C,D) and progression-responsive genes (Figure 3A), we successfully identified 12 consistently upregulated genes (*NPC2*, *F11R*, *ITGAX*, *IL4R*, *CIITA*, *BCL6B*, *TLN1*, *TUBA1C*, *ARHGAP30*, *BCL3*, *FAM60A* and *LRRC32*; Figure 3B) and three consistently downregulated genes (*ZNF252*, *PRB2* and *MAT1A*; Figure 3C). These genes exhibited consistent patterns of expression changes during both cirrhosis progression and HE development, establishing a reliable molecular foundation for subsequent marker gene identification using machine learning.

### 2.4. Identification of Prognosis-Associated DEGs

It has been shown that HE significantly impacts clinical outcomes such as overall survival (OS) in cirrhotic patients [12]. To identify prognosis-associated DEGs, datasets from the prognosis cohort (GSE15654) were subjected to limma-based differential expression analyses (poor prognosis vs. good prognosis) with thresholds *p*-value < 0.05 and |log_2_FC| ≥ 0.5. A total of 412 prognosis-associated DEGs were identified, with 152 upregulated and 260 downregulated (Figure 1 and Figure 3D, Table S1).

Functional enrichment analysis revealed that upregulated DEGs were significantly enriched in pathways regulating cytokine production, cellular development modulation, focal adhesion, and viral infection response (e.g., GO:0001819, positive regulation of cytokine production; GO:0010720, positive regulation of cell development; hsa04510, Focal adhesion; hsa05135, Yersinia infection; Figure S2C and Table S4). Downregulated DEGs were predominantly enriched in small-molecule metabolic pathways (e.g., GO:0044282, small-molecule catabolic process; GO:0044248, cellular catabolic process; GO:0006631, fatty acid metabolic process; Figure S2D and Table S4). This metabolic pattern is consistent with the metabolic suppression characteristic of cirrhosis progression (Figure S2B).

Next, we performed intersection analyses of HE-specific DEGs (Figure 2) and prognosis-associated DEGs, identifying 13 overlapping genes (*MT1X*, *ALOX5*, *PARVG*, *PON3*, *TYROBP*, *SOX9*, *RNASE4*, *C2*, *HAMP*, *SERPINA3*, *CP*, *ANG* and *C1R*; Figure 3E). These genes, associated with both HE and patient survival status, provided good candidates for subsequent HE-specific marker gene selection using machine learning.

### 2.5. Multi-Algorithm Cross-Validated Screening for HE-Specific Marker Genes

To further refine the selection of HE-specific marker genes for model construction, we implemented a machine learning approach using our preliminary findings of (1) 15 shared genes between HE-specific DEGs and cirrhosis progression-reflecting genes (Figure 3B,C), and (2) 13 overlapping genes between HE-specific DEGs and cirrhosis prognosis-associated DEGs (Figure 3E) as input, respectively (Figure 1). Three distinct machine learning algorithms, LASSO (Least Absolute Shrinkage and Selection Operator), RFE (Recursive Feature Elimination), and random forest were used for advanced feature selection across both the progression cohort (GSE139602) and the prognosis cohort (GSE15654; Figure 1).

**Figure 4:**
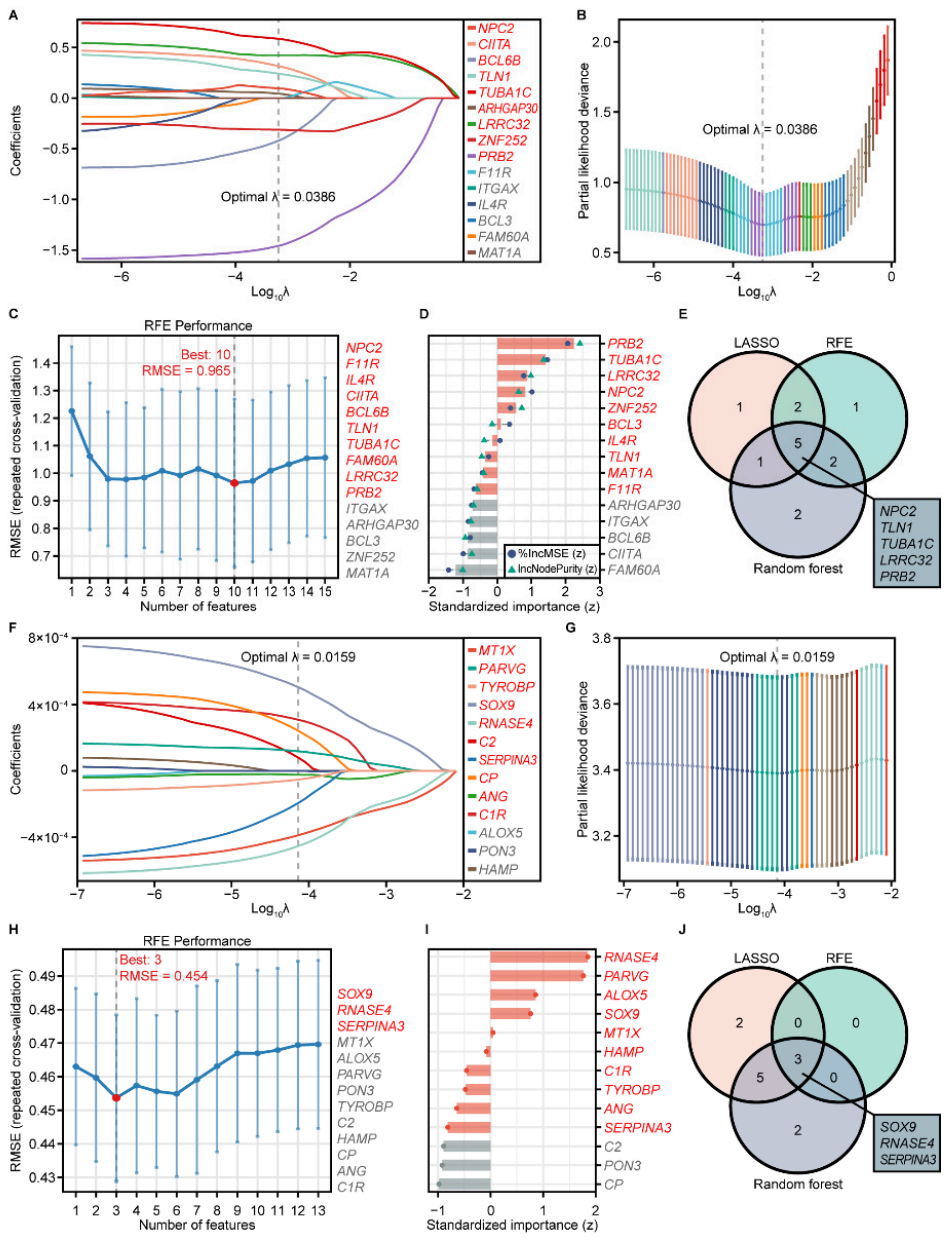
Machine learning-based feature selection in GSE139602 progression cohort and GSE15654 cirrhosis prognosis cohort. (**A**,**B**): LASSO regression analysis showing (**A**) coefficient variation paths and (**B**) 10-fold cross-validation curve (optimal λ = 0.0386), identifying 9 progression-reflecting genes: *NPC2*, *CIITA*, *BCL6B*, *TLN1*, *TUBA1C*, *ARHGAP30*, *LRRC32*, *ZNF252*, and *PRB2*. (**C**) Recursive feature elimination (RFE) analysis determining 10 optimal features based on minimal root mean square error (RMSE): *PRB2*, *TUBA1C*, *CIITA*, *BCL6B*, *IL4R*, *F11R*, *TLN1*, *LRRC32*, *NPC2*, and *FAM60A*. (**D**) Random forest analysis ranking the top 10 most important features by percentage increases in mean squared error (%IncMSE) and node purity (IncNodePurity): *F11R*, *MAT1A*, *TLN1*, *IL4R*, *BCL3*, *ZNF252*, *NPC2*, *LRRC32*, *TUBA1C*, and *PRB2*. (**E**) Venn diagram intersection analysis identifying 5 consensus HE-specific marker genes: *NPC2*, *TLN1*, *TUBA1C*, *LRRC32*, and *PRB2*. (**F**,**G**) LASSO-Cox regression analysis displaying (**F**) coefficient paths and (**G**) partial likelihood deviance curve (optimal λ = 0.0159 with 1 standard error), selecting 10 prognosis-associated genes: *MT1X*, *PARVG*, *TYROBP*, *SOX9*, *RNASE4*, *C2*, *SERPINA3*, *CP*, *ANG*, and *C1R*. (**H**) Survival RFE analysis identifying 3 optimal prognostic genes based on minimal RMSE: *SOX9*, *RNASE4*, and *SERPINA3*. (**I**) Random forest survival analysis ranking the top 10 features by variable importance measures (VIMPs): *SERPINA3*, *ANG*, *TYROBP*, *C1R*, *HAMP*, *MT1X*, *SOX9*, *ALOX5*, *PARVG*, and *RNASE4*. (**J**) Venn diagram intersection analysis identifying 3 consensus HE-specific marker genes: *SOX9*, *RNASE4* and *SERPINA3*.

Within the GSE139602 cohort, the LASSO analysis (λ = 0.0386) selected nine genes (Figure 4A,B), the RFE analysis (minimum RMSE [root mean square error] = 0.965) identified a set of 10 genes (Figure 4C), and the random forest analysis (ranked by a composite of % IncMSE [Percentage Increase in Mean Squared Error] and IncNodePurity [Increase in Node Purity]) captured the top 10 genes (Figure 4D). Five marker genes were consistently selected across all three machine learning algorithms (Figure 4E): *NPC2* (Niemann–Pick disease type C2), *TLN1* (Talin−1, which links integrins to the cytoskeleton), *TUBA1C* (Tubulin Alpha 1C, involved in microtubule assembly), *LRRC32* (Leucine Rich Repeat Containing 32), and *PRB2* (Proline-Rich Protein BstNI Subfamily 2).

In the GSE15654 cohort, LASSO (λ = 0.0159) yielded 10 genes (Figure 4F,G), RFE (minimum RMSE = 0.454) identified three genes (Figure 4H), and random forest (based on feature importance ranking) prioritized the top 10 genes (Figure 4I). Subsequent intersection analysis identified three marker genes (Figure 4J): *SOX9* (SRY-Box Transcription Factor 9), *RNASE4* (Ribonuclease A Family Member 4), and *SERPINA3* (Serpin Family A Member 3).

Collectively, machine learning identified a total of 8 HE-specific marker genes in these two cohorts, with biological characteristics highly congruent with the pathological mechanisms of the liver–brain axis, thereby providing a solid molecular foundation for the construction of early warning models.

### 2.6. Dual-Model Construction Based on XGBoost–SHAP and Multi-Level Clinical Validation

**Figure 5:**
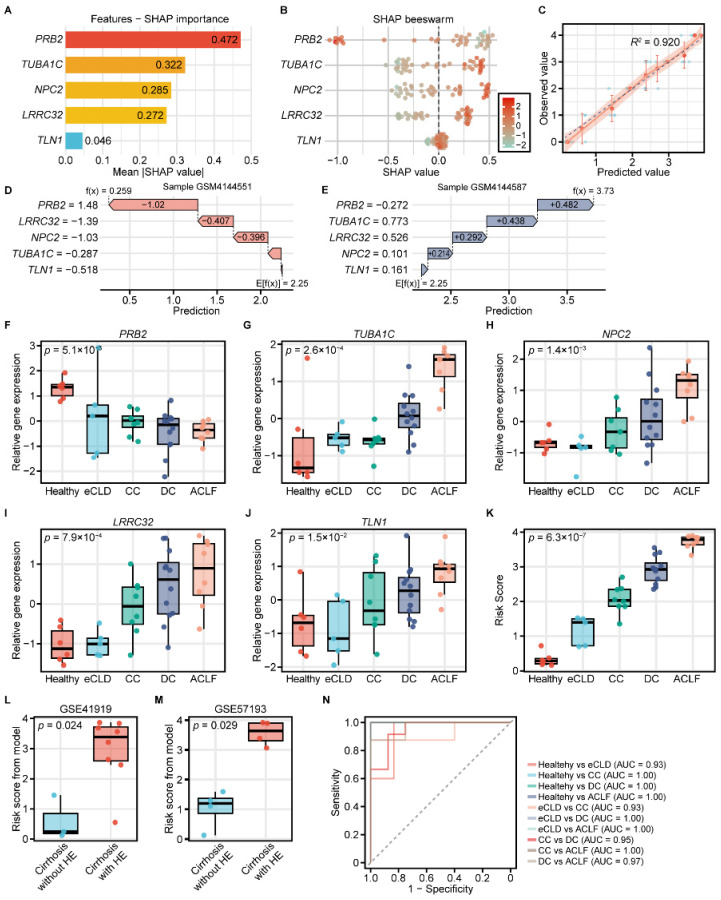
Construction and validation of progression prediction model. (**A**) XGBoost–SHAP analysis showing mean absolute SHAP values of core progression HE-specific marker genes. (**B**) Beeswarm plot displaying individual SHAP values. (**C**) Calibration curve demonstrating model accuracy (*R*^2^ = 0.920). (**D**,**E**) Waterfall plots of individual samples (GSM4144551 and GSM4144587). (**F**–**J**) Significant associations between 5 core HE-specific marker genes and disease progression (*p* < 0.05 considered significant). (**K**) Correlation between risk scores and disease progression. (**L**,**M**) Relationship between risk scores and HE status in validation cohorts (GSE41919 and GSE57193). (**N**) ROC curve analysis of different stages of cirrhosis samples in GSE139602.

Next, we utilized the eXtreme Gradient Boosting (XGBoost) algorithm, in conjunction with SHapley Additive exPlanations (SHAP) analysis, to construct the cirrhosis progression-associated HE risk prediction model based on the progression cohort GSE139602. The eight machine learning-selected HE-specific marker genes were used as input (Figure 4E,J). SHAP analyses delineated the hierarchical contribution of the top five marker genes in the following order: *PRB2* > *TUBA1C* > *LRRC32* > *NPC2* > *TLN1* (Figure 5A). Furthermore, the elevated *PRB2* expression was strongly associated with increased disease risk (negatively correlated with SHAP values), whereas *TUBA1C*, *LRRC32*, *NPC2*, and *TLN1* exhibited protective effects (positively correlated with SHAP values) (Figure 5B). Model validation using the prognosis cohort (GSE15654) exhibited good performance for HE prediction, with a calibration curve *R*^2^ at 0.920 (Figure 5C).

Notably, the sample GSM4144551 (derived from a patient with advanced-stage cirrhosis and identified as high-risk for progression) and the sample GSM4144587 (from a patient with early-stage cirrhosis and identified as low-risk for progression) exhibited the highest SHAP values for *PRB2*, indicating that *PRB2* plays a core regulatory role across different risk groups (Figure 5D,E). Expressions of all five marker genes were significantly associated with disease progression (Figure 5F–J), indicating the biological relevance of these marker genes. Consistently, the model-derived risk score showed a significant correlation with the progression of cirrhosis (Figure 5K), and validation using the two independent HE cohorts (GSE41919 and GSE57193) confirmed that the model maintained robust predictive performance (Figure 5L,M). Additionally, ROC (Receiver Operating Characteristic) analysis evaluating the model’s predictive performance for cirrhosis progression in GSE139602 showed that it could effectively discriminate patients at different stages of cirrhosis (Figure 5N).

**Figure 6:**
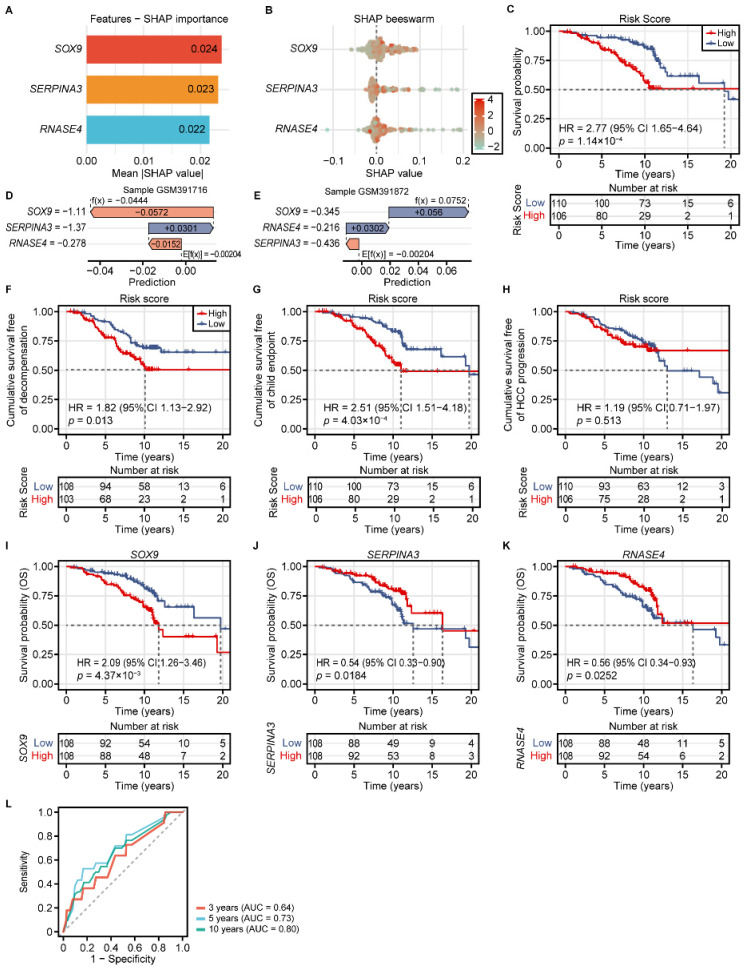
Development and validation of prognostic prediction model. (**A**) XGBoost–SHAP analysis of core prognostic HE-specific marker genes’ contributions. (**B**) Beeswarm plot of individual SHAP values. (**C**) KM survival analysis (overall survival) of risk stratification. (**D**,**E**) Waterfall plots for representative samples (GSM391716 and GSM391872). (**F**–**H**) Multi-endpoint validation showing hepatic decompensation progression (**F**), Child–Pugh deterioration (**G**), and HCC progression (**H**) between risk groups. (**I**–**K**) KM survival analyses (overall survival) validating prognostic value of *SOX9* (**I**), *SERPINA3* (**J**), and *RNASE4* (**K**). (**L**) ROC curve analyses at subsequent years 3, 5, and 10 in the GSE15654 dataset.

To construct the cirrhosis survival-associated HE risk prediction model, we performed XGBoost–SHAP analysis using the prognosis cohort GSE15654 (Figure 1). *SOX9*, *SERPINA3*, and *RNASE4* were identified as key prognostic marker genes (Figure 6A). Consistently, high *SOX9* expression was associated with increased prognostic risk (positively correlated with SHAP values), whereas low expression of *SERPINA3* and *RNASE4* was associated with elevated risk (negatively correlated with SHAP values) (Figure 6B).

Using the median risk score as the cutoff, patients in the cohort GSE15654 were stratified into high-risk and low-risk groups. Kaplan–Meier survival analysis demonstrated that the low-risk group had significantly prolonged overall survival compared with the high-risk group (log-rank *p* < 0.001; Figure 6C). SHAP analysis showed that *SOX9* consistently exhibited the highest feature importance values in both high-risk (e.g., sample GSM391716, derived from a patient with poor prognosis) and low-risk (e.g., sample GSM391872, derived from a patient with favorable prognosis) subgroups (Figure 6D,E), suggesting its pivotal regulatory role regardless of risk classification.

Multi-endpoint clinical validation revealed that the low-risk group had markedly lower risks of progression to hepatic decompensation (*p*-value < 0.05, Figure 6F) and Child–Pugh class deterioration (*p*-value < 0.001, Figure 6G), which is a clinical scoring system used to evaluate hepatic reserve function in patients with cirrhosis [22]. No significant difference between the two groups was found in the risk of developing hepatocellular carcinoma (HCC) (Figure 6H). Univariate survival analysis showed that patients, stratified by median expression, had distinct outcomes: the low-*SOX9* group had better prognosis (*p*-value < 0.05, Figure 6I), while elevated *SERPINA3* and *RNASE4* were also associated with favorable outcomes (*p*-value < 0.05, Figure 6J,K). These stratifications also revealed significant differences in risks assessed by Child–Pugh class deterioration (Figure S3). Although individual maker genes showed significant associations with survival (all *p*-value < 0.05), their combined multi-gene risk score achieved the lowest *p* value (*p*-value < 0.001) by the Kaplan–Meier (KM) analysis between high- and low-risk groups, indicating that the integrated model outperforms single-gene evaluations. ROC analysis was conducted in the GSE15654 cohort to evaluate predictive performance at subsequent years 3, 5, and 10, respectively. The model performed better at years 5 and 10 than at year 3 (Figure 6L).

### 2.7. Structural Equation Modeling-Based Analyses of Characteristic Gene Regulatory Networks

**Figure 7:**
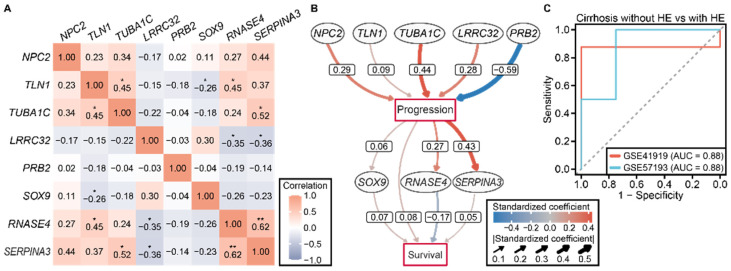
Structural equation modeling. (**A**) Correlation network among 8 key marker genes (red: positive correlation; blue: negative correlation; * *p*-value < 0.05, ** *p*-value < 0.01). (**B**) SEM path analysis (red/blue indicating positive/negative β coefficients). (**C**) ROC curve analysis in cirrhosis-associated HE cohorts (GSE41919 and GSE57193).

We integrated and batch-corrected the progression (GSE139602) and prognosis (GSE15654) cohorts (Figure S4) and examined expression correlations between the two HE marker gene-sets: five HE-specific marker genes (*NPC2*, *TLN1*, *TUBA1C*, *LRRC32*, *PRB2*) and three core prognostic HE-specific marker genes (*SOX9*, *SERPINA3*, *RNASE4*). Co-expression was observed across multiple gene pairs (Figure 7A). For example, *RNASE4* and *SERPINA3* were positively correlated (*r* = 0.62, *p* < 0.05); *TUBA1C* correlated positively with both *RNASE4* (*r* = 0.45) and *TLN1* (*r* = 0.45) but the correlation was weakly negative with *SOX9* (*r* = −0.18, all *p* < 0.05); *LRRC32* showed negative correlations with *SERPINA3* (*r* = −0.36, *p* < 0.01) and *RNASE4* (*r* = −0.35, *p* < 0.05); and *SOX9* was inversely correlated with *TLN1* (*r* = −0.26, *p* < 0.05).

To test whether the eight marker genes relate to disease progression and survival through distinct pathways, we performed structural equation modeling (SEM) integrating gene expressions, progression status, and survival outcomes. Three genes showed direct positive paths to disease progression, namely, *NPC2* (*β* = 0.29, *p*-value < 0.001), *TUBA1C* (*β* = 0.44, *p*-value < 0.01), and *LRRC32* (*β* = 0.28, *p*-value < 0.001), whereas *PRB2* showed a direct negative path (*β* = −0.59, *p*-value < 0.001). Disease progression, in turn, had positive paths to *RNASE4* (*β* = 0.27, *p*-value < 0.001) and *SERPINA3* (*β* = 0.43, *p*-value< 0.001), with indirect effects on survival at −0.0459 and 0.0215, respectively (Figure 7B). The direct path from progression to survival was marginal (*β* = 0.08, *p*-value = 0.22). These results suggest that the two models are not independent but rather interconnected. This prompted us to further integrate both models into a unified framework to better delineate the joint contribution of the eight signature genes to HE outcomes. To quantify the indirect effects of the five progression-associated genes on patient survival through the “from progression status to prognostic genes” axis, we performed mediation decomposition within the SEM framework (Table S5). Notably, none of the indirect effects reached statistical significance for all five genes (all *p*-value > 0.05), suggesting that the impact of these genes on survival operates primarily through direct pathways independent of progression-mediated regulation, rather than through the “from progression status to prognostic genes” cascade.

We combined the two models by AUC-performance weighted fusion of their risk scores (as detailed in Methods, Section 4.6) and validated the integrated score in the HE cohorts (GSE41919 and GSE57193). ROC analysis gave AUC (area under curve) > 0.85 in both datasets (Figure 7C), indicating that combining progression- and prognosis-associated HE-specific marker genes yields a more robust and comprehensive stratification of HE patients than either model in isolation.

### 2.8. GSEA-Based Characterization of Functional Heterogeneity Across HE-Specific Marker Genes

**Figure 8:**
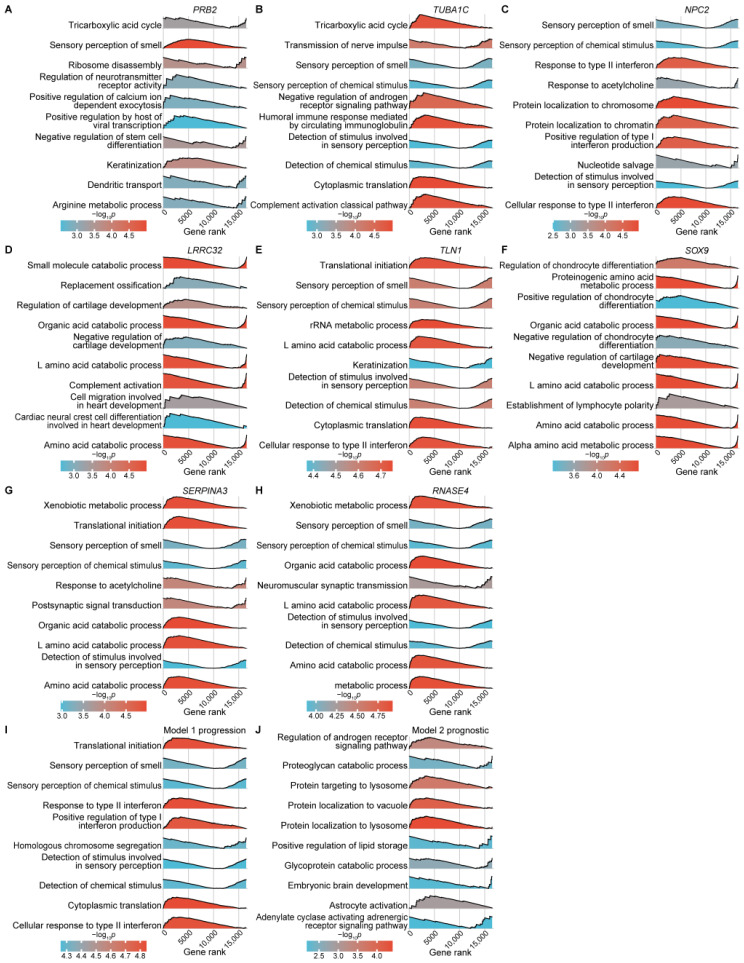
GSEA results for *PRB2* (**A**), *TUBA1C* (**B**), *NPC2* (**C**), *LRRC32* (**D**), *TLN1* (**E**), *SOX9* (**F**), *SERPINA3* (**G**) and *RNASE4* (**H**). Mountain plots display top 5 pathways significantly associated with high (NES > 0) and low (NES < 0) expression groups. (**I**,**J**) GSEA results based on the prediction scores of the progression model (**I**) and the prognostic model (**J**), with top enriched pathways shown for each model.

To dissect the biological functions of the eight core marker genes, we performed single-gene pre-ranked Gene Set Enrichment Analysis (GSEA). These genes were each identified as both a key predictor by the HE-risk model and a mediator of cirrhosis progression or survival outcomes in SEM (Figure 7). Gradient permutation testing (1000, 10,000, and 100,000 permutations) was further applied to validate the robustness of the enrichment results (as detailed in Methods, Section 4.8, and Table S6). The results converged on the following four functional categories:

1. Metabolic homeostasis. *PRB2* and *TUBA1C* were both enriched in the tricarboxylic acid (TCA) cycle, pointing to a role in sustaining energy metabolism (Figure 8A,B). *LRRC32*, *SOX9*, *SERPINA3*, and *RNASE4*, meanwhile, clustered around amino acid and organic acid catabolic processes (Figure 8C–G). These shared metabolic pathways hint at a coordinated effort to preserve the hepatic metabolic capacity.
2. Synaptic and neural function. *PRB2* is mapped to neurotransmitter receptor activity and Ca^2+^-dependent exocytosis, suggesting it protects neural function by modulating synaptic transmission (Figure 8A). *TUBA1C* was linked to chemical stimulus and nerve impulse propagation (Figure 8B), while *NPC2* and *SERPINA3* were associated with sensory perception of chemical stimulus (Figure 8C,G). *RNASE4* enriched the neuromuscular synaptic transmission (Figure 8H). That multiple genes converge on synaptic regulation offers a molecular rationale for the neurological deficits seen in HE.
3. Immune and inflammatory signaling. *NPC2* and *TLN1* showed the strongest enrichment in type II interferon response or type I interferon production (Figure 8C,D), implicating them in interferon-driven immune regulation. *TUBA1C* and *LRRC32* both enriched the complement activation pathways, with *TUBA1C* specifically tied to the classical complement pathway and humoral immunity (Figure 8B,D). These findings align with the well-documented role of systemic inflammation in HE.
4. Detoxification and sensory perception. *SERPINA3* and *RNASE4* jointly activated xenobiotic metabolism, suggesting their potential involvement in hepatic detoxification regulation (Figure 8G,H). Notably, six of the eight genes—*PRB2*, *TUBA1C*, *NPC2*, *TLN1*, *SERPINA3*, and *RNASE4*—enriched the olfactory perception or chemical stimulus detection. Low expression of *SERPINA3* and *RNASE4* was specifically linked to impaired sensory perception, suggesting that metabolic decline may contribute to neurosensory dysfunction in HE.

To further characterize the biological signals captured by the two XGBoost sub-models, we performed GSEA using their prediction scores as phenotypic labels (Figure 8I,J). The progression model score was enriched in translational initiation, cytoplasmic translation, and homologous chromosome segregation—pathways linked to protein synthesis and cell division during cirrhosis progression. In contrast, the prognostic model score was enriched in lysosomal protein targeting, glycoprotein and proteoglycan catabolism, astrocyte activation, embryonic brain development, and androgen receptor regulation. These pathways point to cellular degradation, glial response, neurodevelopmental disruption, and hormonal dysregulation, which may collectively contribute to poor survival in HE patients. Together, these profiles confirm that the two models capture complementary aspects of HE pathogenesis—progression-related translation and chromosomal dynamics versus prognosis-related lysosomal dysfunction, astrocyte activation, and neurodevelopmental impairment.

Together, these results paint a picture of coordinated regulation across metabolism, neurotransmission, immunity, and detoxification, offering mechanistic insights into HE pathogenesis and pointing to potential therapeutic entry points.

## 3. Discussion

Early detection of HE in decompensated cirrhosis remains a major clinical challenge, directly contributing to poor patient outcomes [12,23,24]. Current diagnostic approaches, which depend heavily on neuropsychological assessments and serum ammonia measurement, are hampered by limited sensitivity, operator dependence, and time-consuming procedures [25]. Molecular markers for HE risk stratification remain scarce, even as bioinformatics tools have matured. To address this gap, we combined multi-cohort transcriptomic data with a tri-algorithm machine learning pipeline and identified eight HE-specific marker genes (*PRB2*, *TUBA1C*, *NPC2*, *LRRC32*, *TLN1*, *SOX9*, *RNASE4* and *SERPINA3*).

Leveraging this gene panel, we then constructed an XGBoost-based HE risk prediction system with two complementary sub-models: one for cirrhosis progression (GSE139602 cohort; genes: *PRB2*, *TUBA1C*, *LRRC32*, *NPC2*, and *TLN1*) and one for patient survival (GSE15654 cohort; genes: *SOX9*, *SERPINA3*, and *RNASE4*). For the progression model, calibration was strong (*R*^2^ = 0.920) and discrimination across cirrhosis stages was clear (Figure 5). For the prognostic model, patients stratified by risk showed distinct overall survival between high- and low-risk groups (log-rank *p* < 0.001; Figure 6C). Recognizing that each sub-model captures a distinct dimension of the disease trajectory, we integrated them via AUC-performance weighted fusion. The resulting integrated score achieved AUC > 0.85 in both independent HE validation cohorts (Figure 7C), confirming its robust cross-cohort generalizability.

Among the eight marker genes, *SOX9* has been reported as a biomarker for liver cancer stem cells that promotes HCC proliferation and drives drug resistance [26,27]. Evidence also indicates that *SERPINA3* (mouse homolog: *SerpinA3N*) plays a pivotal pro-inflammatory role in neuroinflammatory processes of Alzheimer’s disease and other neurodegenerative disorders [28,29]. Besides, the relevant literature also reports that this gene is closely associated with liver fibrosis [30]. In contrast, the other six genes (*PRB2*, *TUBA1C*, *NPC2*, *LRRC32*, *TLN1*, and *RNASE4*) have not been linked to liver disease or neurodegenerative disorders before. Although these genes lack prior disease associations, our GSEA suggests that they may contribute to HE through several pathways: metabolic regulation and synaptic function are the strongest hits, while immune signaling and detoxification pathways also appear involved. Because these findings are purely computational, their functional roles in HE need further validation.

Metabolic dysregulation along the gut–liver–brain axis is a key mechanism in HE pathogenesis. Under normal conditions, the liver regulates ammonia detoxification, glycogen homeostasis, and lipid metabolism [31]. When cirrhosis develops, these metabolic pathways are suppressed and detoxification capacity declines. According to the gut–liver–brain axis model, intestinal microbes produce neurotoxic metabolites such as ammonia and indoles, which enter the liver through the portal vein; because cirrhosis-related enzyme deficiencies (e.g., urea cycle dysfunction) reduce clearance, these toxins accumulate and cross the blood–brain barrier, where they activate microglia and disrupt astrocyte and neurotransmitter function [32,33,34]. To test whether this axis-level disruption is reflected at the transcriptomic level, we profiled gene expressions in cirrhotic cohorts. Hepatic detoxification pathways were indeed suppressed, including small-molecule catabolic processes (GO:0044282) and fatty acid metabolism (GO:0006631; Figure 3C), which aligns with the metabolic decompensation predicted by the gut–liver–brain axis model.

The XGBoost–SHAP model confers several distinct advantages over conventional neuropsychological screening. First, by relying on objective transcriptomic markers rather than subjective assessments, it eliminates inter-operator variability and the need for active patient cooperation. Second, the integration of SHAP analysis enhances clinical interpretability by quantifying each gene’s contribution to individual risk predictions, thereby addressing a key barrier to the adoption of machine learning (its “black-box” nature) in clinical practice. Finally, the dual-model framework offers a comprehensive clinical picture, simultaneously assessing both progression risk and survival prognosis, which allows the same eight-gene panel to guide decision-making across the continuum of cirrhosis care.

Nevertheless, several limitations should be acknowledged. All analyses were based on retrospective transcriptomic data, and prospective multicenter studies are required before clinical translation. The functional enrichment findings, while informative, do not establish causation, and require experimental validation. Furthermore, the current model’s dependence on hepatic tissue biopsies limits its immediate utility; future integration of circulating biomarkers could facilitate a non-invasive screening approach. Due to data availability, direct comparisons with established clinical scoring systems (MELD, Child-Pugh, and PHES) or other reported HE biomarkers were not feasible. Finally, the small size of the HE validation cohorts constrains statistical power for subgroup analyses. Importantly, these limitations largely stem from the discovery-oriented nature of this transcriptomic study—our primary goal was to identify molecular signatures and establish a proof-of-concept model rather than to immediately replace existing clinical tools. Within this framework, the biologically interpretable pathways and the predictive framework we have established provide a solid foundation for future prospective validation and clinical translation.

## 4. Materials and Methods

### 4.1. Data Acquisition and Cohort Construction

We curated cirrhosis and HE transcriptomic datasets from the GEO database (Available online: https://www.ncbi.nlm.nih.gov/geo/, accessed on 3 December 2025) using the keywords “cirrhosis” and “hepatic encephalopathy”. To assess sample heterogeneity, we performed hierarchical clustering (“stats” package, https://search.r-project.org/R/refmans/stats/html/00Index.html, v4.3.3, accessed on 29 July 2026) and principal component analyses (prcomp function, visualized with “ggplot2”, https://CRAN.R-project.org/package=ggplot2, v4.0.1, accessed on 29 July 2026). Based on this assessment, samples were included if they had valid group labels (cirrhosis/healthy controls or HE/non-HE) and complete clinical data, particularly prognostic parameters. Four cohorts met these criteria: GSE41919 (3 cirrhosis without HE, 8 cirrhosis with HE), GSE57193 (4 cirrhosis without HE, 4 cirrhosis with HE), GSE139602 (6 healthy controls, 5 eCLD, 12 CC, 8 DC, 8 ACLF), and GSE15654 (216 cirrhosis samples). From these cohorts, dataset acquisition, gene annotation, and clinical metadata extraction were performed using the “GEOquery” package (https://github.com/seandavi/GEOquery, v2.70.0, accessed on 29 July 2026) in R (https://www.r-project.org/, v4.3.3, accessed on 29 July 2026).

### 4.2. Differential Expression Analysis

Differential expression analysis was conducted utilizing the “limma” package [35] (v3.58.1) in R. The workflow consisted of linear model fitting (lmFit), contrast specification (contrasts.fit), and empirical Bayes moderation (eBayes) for variance stabilization. Genes with adjusted *p*-value < 0.05 and |log_2_FC| ≥ 0.5 were considered differentially expressed and visualized as volcano plots using “ggplot2”. To identify shared transcriptional changes, we then intersected the differentially expressed genes from GSE41919 and GSE57193 and displayed the overlap using “ggvenn” (https://CRAN.R-project.org/package=ggvenn, v0.1.19, accessed on 29 July 2026). All analyses were run in R with predetermined random seeds.

### 4.3. Functional Enrichment Analysis

Functional enrichment analysis was performed on the differentially expressed gene clusters using the “clusterProfiler” package [36] (v4.10.1). Both GO terms and KEGG pathways were tested. The GO terms covered Biological Process (BP), Molecular Function (MF), and Cellular Component (CC). Gene identifiers were first converted using the “bitr” function, after which enrichment was calculated separately with “enrichGO” and “enrichKEGG”. All *p*-values were adjusted using the Benjamini–Hochberg (BH) method, with significance set at adjusted *p* < 0.05. Enrichment results were then visualized as dot plots using “ggplot2”.

### 4.4. Identification of Dynamically Responsive Genes

Gene expression analysis was performed across disease progression stages in the GSE139602 cohort; mean expression values were computed and then analyzed by soft clustering using the “Mfuzz” package (https://bioconductor.org/packages/devel/bioc/html/Mfuzz.html, v2.62.0, accessed on 29 July 2026). Fuzzy C-means clustering was applied to identify gene expression patterns, with parameters including a fuzzification coefficient (m-value) of 1.99 (determined via the “mestimate” function) and an optimal cluster number c = 12. Cluster-specific expression dynamics were plotted using “mfuzz.plot2”, showing centroid trajectories across disease stages. Next, dynamically responsive genes were selected based on module association (correlation coefficient > 0.4) and monotonic expression profiles (sustained upregulation or progressive downregulation throughout disease advancement). Finally, these gene sets were subjected to GO/KEGG pathway enrichment analyses.

### 4.5. Machine Learning-Based Marker Gene Selection

Marker gene selection was performed using transcriptomic data from two clinical cohorts: GSE139602 (cirrhosis progression) and GSE15654 (cirrhosis prognosis). In the progression cohort, three methods were applied. LASSO regression was performed using the “glmnet” package [37,38] (v4.1–10), with optimal λ.min determined via 10-fold cross-validation. RFE was performed using the “caret” package (https://CRAN.R-project.org/package=caret, v7.0–1, accessed on 29 July 2026) to minimize RMSE through backward selection. Random forest modeling was conducted using the “randomForest” package (https://CRAN.R-project.org/package=randomForest, v4.7–1.2, accessed on 29 July 2026), with variable importance evaluated by “%IncMSE” and “IncNodePurity”. For the prognosis cohort, survival-oriented methods were used: LASSO–Cox proportional hazards modeling with “glmnet”, survival-adapted RFE with “caret”, and survival random forest analysis using the “randomForestSRC” package (https://CRAN.R-project.org/package=randomForestSRC, v3.5.0, accessed on 29 July 2026), with its variable importance quantified by variable importance measures (VIMP). The marker genes panel was then derived from the intersection of features shared by all three methods within each cohort.

### 4.6. Construction and Validation of XGBoost–SHAP Models

The selected HE-specific marker genes were used to develop two models: a cirrhosis progression classifier and a prognostic risk stratification model. In the GSE139602 progression cohort, an XGBoost regression model (“xgboost” package [39] v3.1.3.1) was trained for multiclass prediction, with hyperparameters including: “reg:squarederror” objective function, RMSE evaluation criterion, learning rate (η = 0.05), maximum tree depth (d = 3), subsampling ratio (0.7), and L2 regularization (λ = 5). Training incorporated 5-fold cross-validation with early stopping (patience = 50 epochs). For the GSE15654 prognosis cohort, an XGBoost survival model was trained using the “survival:cox” objective function and “cox-nloglik” evaluation metric, with the parameters being learning rate (η = 0.05), maximum depth (d = 6), subsampling ratio (0.9), colsample_bytree = 1, and L2 regularization (λ = 0.5). As with the progression model, 5-fold cross-validation with early stopping (50 epochs) was applied.

SHAP values were calculated using the “shapviz” package (https://github.com/ModelOriented/shapviz, v0.10.3, accessed on 29 July 2026) and visualized as beeswarm plots showing the relationship between feature values and SHAP values. For model validation, the progression model was evaluated using probability calibration curves (“ggplot2” package). The prognostic model was assessed using KM survival analysis (“survminer” package, https://CRAN.R-project.org/package=survminer, v0.5.1, accessed on 29 July 2026); patients were stratified by the median risk score, with significance set at *p*-value < 0.05.

To integrate the two sub-models, we constructed an integrated risk score based on AUC-performance weighting. The fusion weights were determined by the proportion of each model’s AUC in the validation cohorts (w_1_ = AUC_1_/(AUC_1_ + AUC_2_); w_2_ = AUC_2_/(AUC_1_ + AUC_2_)). Risk scores were Z-score-standardized and then weighted-summed.

### 4.7. Structural Equation Modeling and Mediation Effect Analysis

Gene expression data from both the GSE139602 and GSE15654 cohorts were analyzed jointly. Batch effects were corrected using the “ComBat” algorithm (“sva” package, https://bioconductor.org/packages/release/bioc/html/sva.html, v3.50.0, accessed on 29 July 2026). After batch correction, normality was assessed using the Shapiro–Wilk test (“stats” package); Pearson correlation was then applied for normally distributed genes/samples, and Spearman’s rank correlation for non-normally distributed ones. Correlations are reported as r values with two-tailed *p*-values, with significance set at *p* < 0.05. For SEM, the “lavaan” package (https://CRAN.R-project.org/package=lavaan, v0.6–21, accessed on 29 July 2026) was used with parameters estimated by maximum likelihood. Path coefficients were validated by bootstrap resampling (B = 2000).

To dissect the direct and indirect effects of individual genes within the regulatory axis from gene expression to disease progression to patient survival, we performed mediation analysis within the SEM framework. The total effects of the five progression-associated genes (*NPC2*, *TLN1*, *TUBA1C*, *LRRC32*, and *PRB2*) on survival were decomposed into: (1) direct effects from each gene to survival, not mediated by progression status; and (2) indirect effects from each gene to progression status, then to prognostic genes, and finally to survival, mediated via progression status and downstream prognostic genes. A bias-corrected percentile bootstrap with 2000 resamples was used to estimate confidence intervals for indirect effects.

### 4.8. Gene Set Enrichment Analysis (GSEA)

Single-gene pre-ranked GSEA was performed. All genes were first ranked by Spearman’s rank correlation coefficients (Spearman’s *r*) relative to the HE-specific marker genes in descending order of their expressions. GO/Biological Process (GO BP) terms (*n* = 7168) were then extracted from MSigDB C5 using the “msigdbr” package (https://CRAN.R-project.org/package=msigdbr, v25.1.1, accessed on 29 July 2026). Enrichment analysis was performed using the “fgsea” package (https://github.com/alserglab/fgsea, v1.37.4, accessed on 29 July 2026), with gene set size thresholds (minSize = 15, maxSize = 500) and FDR (false discovery rate) < 0.05 after BH correction (or *p*-value < 0.01). To validate the robustness of the enrichment results, gradient permutation testing with increasing iteration counts (1000, 10,000, and 100,000 permutations) was applied to both single-gene and model-score-based analyses (Table S6). The overlap of significant pathways between adjacent gradient levels was evaluated, with 1000 permutations identified as insufficient and 100,000 permutations adopted for final analyses to ensure stability. Finally, the top positively enriched (NES > 0) and negatively enriched (NES < 0) pathways were identified by absolute NES and plotted using “ggplot2”.

### 4.9. Statistical Analysis and Data Visualization

Continuous variables were compared between groups using distribution-appropriate tests: independent two-sample *t*-tests (“stats” package) for normally distributed data, and the Mann–Whitney U test (“stats” package) for non-normally distributed data. Categorical variables were compared using Pearson’s chi-square test (“stats” package). Survival outcomes were plotted as KM curves (“survminer” package) and compared using log-rank tests (“survival” package, https://CRAN.R-project.org/package=survival, v3.8–3, accessed on 29 July 2026). ROC curve analysis was performed using the “pROC” package (https://CRAN.R-project.org/package=pROC, v1.19.1.0, accessed on 29 July 2026) and visualized with “ggplot2”. All analyses were performed in R, using “ggplot2”, “dplyr” (https://CRAN.R-project.org/package=dplyr, v1.1.4, accessed on 29 July 2026), “cowplot” (https://CRAN.R-project.org/package=cowplot, v1.2.0, accessed on 29 July 2026), “ggplotify” (https://CRAN.R-project.org/package=ggplotify, v0.1.3, accessed on 29 July 2026), and “gridExtra” (https://CRAN.R-project.org/package/gridExtra, v2.3, accessed on 29 July 2026) for visualization and data manipulation. Significance was set at *p*-value < 0.05 for all tests.

To ensure the reproducibility of this study, all code used in analysis has been organized and made available on GitHub (https://github.com/yuanfeng-lan/HE-risk-Prediction, accessed on 29 July 2026), with detailed annotations and an environment configuration file. The code covers data preprocessing, normalization approaches, model training and validation, hyperparameter tuning, cross-validation strategies, random seed settings, statistical testing, and visualization.

## Figures and Tables

**Table 1:** Datasets used in this study.

Dataset	Samples	Meta Data	Reference
GSE41919	3 cirrhosis samples without HE and 8 cirrhosis samples with HE	-	PMID: 31571389
GSE57193	4 cirrhosis samples without HE and 4 cirrhosis samples with HE	-	PMID: 31571389, PMID: 25064044
GSE139602	6 healthy controls, 5 early chronic liver disease cases (eCLD), 12 compensated cirrhosis specimens (CC), 8 decompensated cirrhosis samples (DC), and 8 acute-on-chronic liver failure samples (ACLF)	-	PMID: 35540106, PMID: 40437093
GSE15654	216 cirrhosis samples	Prognostic information	PMID: 23333348, PMID: 28256512, PMID: 31344396

## Data Availability

All data utilized in this study were obtained from the Gene Expression Omnibus (GEO) database, a publicly accessible repository maintained by the National Center for Biotechnology Information (NCBI). All datasets are freely available for download without any access restrictions. The specific GEO accession numbers and corresponding datasets are cited within the manuscript.

## Supplementary Materials

The following supporting information can be downloaded at: https://www.mdpi.com/article/10.3390/ijms27156925/s1.
